# Microbial Surface Biofilm Responds to the Growth-Reproduction-Senescence Cycle of the Dominant Coral Reef Macroalgae *Sargassum* spp.

**DOI:** 10.3390/life11111199

**Published:** 2021-11-06

**Authors:** Bettina Glasl, Jasmine B. Haskell, Tania Aires, Ester A. Serrão, David G. Bourne, Nicole S. Webster, Pedro R. Frade

**Affiliations:** 1 Centre for Microbiology and Environmental Systems Science, Division of Microbial Ecology, University of Vienna, 1030 Vienna, Austria; 2 CCMAR-Centre of Marine Sciences, CIMAR, University of Algarve, 8005-139 Faro, Portugal; a62825@ualg.pt (J.B.H.); taires@ualg.pt (T.A.); eserrao@ualg.pt (E.A.S.); 3 Australian Institute of Marine Science, Townsville 4810, Australia; 4 College of Science and Engineering, James Cook University, Townsville 4811, Australia; david.bourne@jcu.edu.au; 5 Australian Centre for Ecogenomics, University of Queensland, Brisbane 4072, Australia; 6 Australian Antarctic Division, Hobart 7050, Australia; nicole.webster@awe.gov.au; 7 Zoological Department III, Natural History Museum Vienna, 1010 Vienna, Austria; pedro.frade@nhm-wien.ac.at

**Keywords:** macroalgae microbiome, microbialization, 16S rRNA gene amplicon sequencing, metagenome assembled genomes, coral reefs

## Abstract

Macroalgae play an intricate role in microbial-mediated coral reef degradation processes due to the release of dissolved nutrients. However, temporal variabilities of macroalgal surface biofilms and their implication on the wider reef system remain poorly characterized. Here, we study the microbial biofilm of the dominant reef macroalgae *Sargassum* over a period of one year at an inshore Great Barrier Reef site (Magnetic Island, Australia). Monthly sampling of the *Sargassum* biofilm links the temporal taxonomic and putative functional metabolic microbiome changes, examined using 16S rRNA gene amplicon and metagenomic sequencing, to the pronounced growth-reproduction-senescence cycle of the host. Overall, the macroalgal biofilm was dominated by the heterotrophic phyla *Firmicutes* (35% ± 5.9% SD) and *Bacteroidetes* (12% ± 0.6% SD); their relative abundance ratio shifted significantly along the annual growth-reproduction-senescence cycle of *Sargassum*. For example, *Firmicutes* were 1.7 to 3.9 times more abundant during host growth and reproduction cycles than *Bacteroidetes*. Both phyla varied in their carbohydrate degradation capabilities; hence, temporal fluctuations in the carbohydrate availability are potentially linked to the observed shift. Dominant heterotrophic macroalgal biofilm members, such as *Firmicutes* and *Bacteroidetes*, are implicated in exacerbating or ameliorating the release of dissolved nutrients into the ambient environment, though their contribution to microbial-mediated reef degradation processes remains to be determined.

## 1. Introduction

A consistent feature of many degraded coral reefs is the shift from a coral- to an algal-dominated benthos [1,2,3], which is also associated with a dramatic reduction in coral biodiversity and loss of reef ecosystem resilience [4,5]. Corals and algae, as primary producers in coral reefs, exudate dissolved organic carbon (DOC) into the ambient seawater, which goes on to support microbial life [6,7,8]. Microbes play a fundamental role in the underlying mechanism of spatial competition between algae and corals on the reef benthos [6,7,9,10]. However, the quantity of DOC released by turf and macroalgae is significantly higher and compositionally different than that released by corals, consequently generating an excess amount of algal-derived DOC [6,9]. Algal-derived DOC stimulates an increase in the planktonic microbial biomass and energy usage through the growth of copiotrophic microbes [8,9,11], which in return can lead to an increase in coral mortality [6,7,12,13]. This microbe-mediated coral mortality frees space on the reef benthos, facilitating further algal growth [9]. This positive feedback loop of DOC, disease, algae and microbes is referred to as the DDAM loop [9,10]. While the interplay between macroalgae proliferation and the microbialization of coral reef seawater is well understood, less is known about the compositional and functional dynamics of the macroalgal-associated microbial biofilm and its interrelations to changes in the reef ecosystem.

Macroalgal health, nutrition, and development are strongly linked to its associated microbiome [14,15], with the host and its microbial partners forming a tightly coupled holobiont [16,17]. A variety of bacteria, archaea, viruses, and eukaryotic microorganisms such as microalgae, protozoa and fungi comprise the algal holobiont [18]. Host-associated microbial biofilms act as an interface between the host and the ambient environment and thus are responsible for the exchange of energy, nutrients and metabolites [15]. Bacteria associated with macroalgal biofilms modulate key functions such as protection against pathogens, nutrient provisioning, morphogenesis, reproduction, and zoospore settlement [18,19,20,21,22]. In return, the algal hosts provide their bacteria with an oxic environment, a habitable substrate, and nutrition [23,24]. Due to the unique position of macroalgal biofilms on the interface of the host with its surrounding environment, they can provide valuable information regarding the health of benthic macroalgae and external environmental conditions [18,25]. Hence, it is important to understand how the macroalgal-associated microbiome varies throughout the life stages of the host and in response to environmental fluctuations.

Algal-associated microbial communities vary over time, space, and among macroalgal host species [14,26,27,28,29]. Abiotic factors such as light availability, wave energy, nutrient concentrations, and temperature can alter macroalgal bacterial biofilm community composition in temperate and subtropical regions [29,30,31,32]. For example, external nitrogen deficiency stress in *Sargassum horneri* was correlated with a dominance of microbes performing nitrogen transporting functions compared to unaffected *S. horneri* [24]. However, the effects of increased environmental stressors related to a warming climate on the macroalgae’s relationship with its associated microbiome remain uncertain [18].

*Sargassum*, a canopy-forming brown algae, is a vital primary producer and ecosystem engineer [14]. Similar to other macroalgae, *Sargassum* provides structure to the benthos and food for organisms across multiple trophic levels [33]. However, high-density blooms of macroalgae can negatively impact coral larval recruitment and metamorphosis, juvenile coral growth and survival, and coral health [2,34,35,36] through direct factors such as shading, abrasion, and space occupation [37,38]. Macroalgae have also been implicated as potential vectors for the increase in disease-associated microbes in corals [13,39,40]. On Australia’s Great Barrier Reef (GBR), some highly seasonal *Sargassum* species dominate inshore reef habitats [41,42,43], with their growth, reproduction and senescence tightly linked to changes in seawater temperature [44]. For example, at macroalgae-dominated inshore GBR locations, *Sargassum* grows rapidly from October to February and reaches its peak biomass at around April [43,45,46]. At Magnetic Island (our study site), in the Central GBR, the highest densities of reproductive *Sargassum* thalli have previously been observed between January and April; hence, the reproductive phase coincides with the peak in biomass [47]. In late May to June, *Sargassum* undergoes senescence and annual tissues start to degenerate, while the remaining thalli can be heavily epiphytized [44,47,48]. In August, the peak of austral winter, the *Sargassum* biomass is reduced to its minimum [43,45,49].

In this study, we investigate the taxonomic and functional dynamics of the surface biofilm microbiome of the macroalgae *Sargassum* spp. (including ‘stem’, blades, and vesicles) over a period of one year to cover the entire annual growth-reproduction-senescence cycle. Furthermore, potential links between shifts in the bacterial community composition (using 16S rRNA gene amplicon sequencing) and fluctuations in abiotic environmental parameters (measured *in situ*) are analyzed using an array of multivariate statistical approaches. The underlying functions of keystone bacterial taxa are further assessed using comparative metagenomics.

## 2. Materials and Methods

### 2.1. Sample Collection and Processing

The samples were collected as part of the Australian Microbiome Initiative and the sample procedure and preparation has previously been outlined in Glasl et al. [50,51]. In brief, macroalgae samples along with environmental metadata (including sediment and seawater data) were collected at 10 time points distributed over one year (in 2016, samples were collected in February, March, April, June, August, October, November, and December; in 2017, samples were collected in February and March) at Geoffrey Bay, Magnetic Island (Great Barrier Reef, Queensland, Australia).

At least four highly similar *Sargassum* species with identical life-cycle patterns have been described to occur on the reefs surrounding Magnetic Island [47]. Macroalgae samples have been identified to genus level; thus, we are referring to the samples as *Sargassum* spp. ‘Stem’, blades, and vesicles of *Sargassum* spp. were sampled at 3 m depth using sterile scalpel blades, rinsed with 0.2 µm filter-sterilized seawater to remove loosely attached microbes, snap frozen in liquid nitrogen, and stored at −80 °C. The *Sargassum* spp. biofilm was subsequently removed from the macroalgal tissue by overnight incubation at 200 rpm in 10 mL 1× PBS at 37 °C and the suspended biofilm was pelleted by a 10 min centrifugation at 16,000 rcf at 4 °C. The biofilm pellet was used for DNA extractions following the manufacturer’s instructions using the DNeasy PowerSoil kit (QIAGEN).

Seawater samples for environmental metadata were collected with a diver-operated Niskin bottle at 2 m depth. The abiotic seawater metadata included: dissolved inorganic nutrients (ammonium, nitrate, phosphate), total suspended solids (TSS), chlorophyll *a* concentration (Chla), salinity, particulate nitrogen (PN), total nitrogen (TN), non-purgeable organic carbon (NPOC), non-purgeable inorganic carbon (NPIC), and silica. A 100 mL glass jar was used for sediment collection at 2 m depth. Total organic carbon (TOC) and grain size distribution was determined for the sediment samples. Environmental metadata were analyzed following the standardized procedures by the Australian Institute of Marine Science (AIMS) [52]. Seawater temperatures were extracted from AIMS long-term monitoring temperature records (https://data.aims.gov.au/). All samples were collected under the permit G16/38348.1 issued by the Great Barrier Reef Marine Park Authority.

### 2.2. 16S rRNA Gene Sequencing Data

All *Sargassum* spp. biofilm DNA extracts (n = 3 samples per sampling event, total of 30 samples) were used for 16S rRNA gene amplicon sequencing. The sequencing data were previously included as part of a large-scale reef ecosystem analysis and a detailed description of the read processing has been outlined in Glasl et al. [50]. In brief, bacterial 16S rRNA gene amplicon sequencing of an approximately 500 bp segment of the hypervariable region V1-V3 was conducted at the Ramaciotti Centre for Genomics (Sydney, Australia). Following the standard operational procedures of the Australian Microbiome Initiative (https://www.australianmicrobiome.com), the bacterial 16S rRNA gene was sequenced using the 27F [53] and 519R [54] primers on the Illumina MiSeq platform following a dual index 2 × 300 bp paired-end approach. The 16S rRNA gene sequencing data were analyzed as zero-radius operational taxonomic units (zOTU) following the standardized analysis platform of the Australian Microbiome Initiative [50,55]. In brief, paired-end reads were merged using FLASH software [56] and FASTA formatted sequences were extracted from FASTQ files. Sequences < 400 bp in length, and/or containing one or more Ns, or homopolymer runs of >8 bp were removed with MOTHUR v1.34.1 [57]. Using USEARCH 64 bit v10.0.240 [58], sequences were de-replicated, ordered by abundance and sequences with less than four representatives and Chimeras were removed. The quality-controlled sequences were mapped to zOTUs. A zOTU table containing samples and read abundances was created. The taxonomy of zOTUs was classified with the SILVA v132 database [59] using MOTHUR’s implementation of the Wang classifier [60] and a 60% Bayesian probability cut-off. Chloroplast- and mitochondria-derived reads were removed from the final dataset.

Raw amplicon sequencing data, zOTU tables, and metadata are freely available at the Bioplatforms Australia data portal under the Australia Microbiome Initiative (https://data.bioplatforms.com/organization/about/australian-microbiome). Full usage requires free registration. To search for the 16S rRNA sequencing data, navigate to ‘Processed data’, select ‘Amplicon is 27f519r_bacteria’ and ‘Environment is Marine’. To search for *Sargassum* spp. samples, add an additional contextual filter, select ‘Sample Site Location Description’ from the dropdown menu and search for ‘Geoffrey Bay’ and ‘Sample Type is Seaweed’.

### 2.3. Metagenome-Assembled Genomes (MAGs)

A subset of the DNA extracts (n = 6, collected in August 2016 and February 2017, the peaks of winter and summer, respectively) were sent for metagenome sequencing to the Australian Genome Research Facility (AGRF, Melbourne, Australia). Metagenome sequencing, assembly, and binning has been described in detail in Glasl et al. [51]. Briefly, libraries for metagenome sequencing were prepared with the Nextera Library Preparation Kit (Illumina) and sequenced on a HiSeq 2500 in rapid run mode with 250 bp paired-end reads. Quality controlled metagenome reads were used to generate MAGs with uniteM v0.0.15 (https://github.com/dparks1134/UniteM). The taxonomy of the retrieved MAGs was assigned using GTDBtk v0.2.1 (https://github.com/Ecogenomics/GTDBTk) and functional annotation was performed with enrichM v0.4.7 (https://github.com/geronimp/enrichM) using the Kyoto Encyclopedia of Genes and Genomes Orthology (KEGG; KOs) as well as the Carbohydrate Active enzyme (CAZy) database (Figures S1 and S2). The presence of KOs in each MAG was assessed for carbohydrate metabolism (i.e., central carbohydrate metabolism) and energy metabolism (i.e., carbon fixation, nitrogen metabolism, and sulfur metabolism). Furthermore, using the CAZy database, each MAG was screened for the presence of Glycoside Hydrolases (GH), Glycosyl Transferases (GT), Polysaccharide Lyases (PL), and Carbohydrate Esterases (CE). The 20 high-quality MAGs can be retrieved from the NCBI BioProject PRJNA594068.

### 2.4. Statistical Analyses of Microbial Community Composition

The 16S rRNA gene sequence data were rarefied to the minimum number of reads per sample (6800). The rarefied dataset was used for the statistical analyses with the exception of the DESeq2 analysis [61], where non-rarefied count data were used. Relative abundance data were used to generate the bubble plot and correlograms. Statistical analyses were performed in R [62] using phyloseq [63], vegan [64], DESeq2 [61], ggbiplot available online: http://github.com/vqv/ggbiplot, GGally available online: https://github.com/ggobi/ggally, microbiomeSeq available online: https://github.com/umerijaz/microbiomeSeq, and graphical package ggplot2 [65] unless otherwise stated.

To analyze variations in microbial community composition along the annual growth-reproduction-senescence cycle of *Sargassum* spp., samples were grouped based on their sampling date into the following three life stages: (i) ‘growth’ (October-December), (ii) ‘reproduction’ (February-April), and (iii) ‘senescence’ (June-August). Grouping was based on previously published studies on the life cycle stages of *Sargassum* spp. in close proximity to the study site [43,45,46,47] and was further confirmed by personal observations.

Three alpha diversity indices were calculated for this study: Shannon index, Chao1 and Observed zOTUs. zOTUs with one count (singletons) were kept for alpha diversity analysis but were removed for subsequent analyses. A Venn diagram, using singleton-depleted count data, was generated and represents the unique, shared, and ubiquitous zOTUs among the three life stages (‘growth’, ‘reproduction’, and ‘senescence’). The beta diversity was assessed using non-metric multidimensional scaling (nMDS) based on binary Bray–Curtis dissimilarity matrices. Differences in the microbial community composition among life stages (i.e., ‘growth’, ‘reproduction’, and ‘senescence’) were further assessed using a permutational multivariate analysis of variance (PERMANOVA). Heterogeneity in sample dispersions among the three life stages was tested using a permutational analysis of multivariate dispersion (PERMDISP). Significance values (*p*-values) of PERMANOVA and PERMDISP were computed with 1000 permutations and adjusted with the Bonferroni method. Normality and homogeneity of variances for alpha diversity measures and the environmental parameters were tested using quantile and density distribution plots in conjunction with the Shapiro–Wilks test. Alpha diversity measures required inverse and square root transformations. Environmental metadata only required an inverse transformation. Parametric or non-parametric methods were implemented accordingly. The relationship of sampling time points and environmental parameters was assessed with principal component analysis (PCA) based on Euclidean distances. A correlogram generated with GGally was plotted to identify and remove collinear variables. Variables with a Pearson correlation lower than −0.7 or higher than 0.7 were removed from subsequent analysis and environmental parameters were scaled to account for their varying units.

A distance-based redundancy analysis (db-RDA) followed by a variation partitioning analysis was conducted to investigate the link between the biofilm community and the environmental parameters. The ordiR2step function of the vegan package was used to generate models based on the db-RDA. The fit of the models and the included environmental parameters was assessed with a one-way analysis of variance (ANOVA). Abiotic factors deemed relevant by the db-RDA were categorized into ‘high’ and ‘low’ based on their median values (cut-off values for temperature = 27 °C, NPOC = 1.33 mg/L, NH_4_^+^ = 0.22 µmol/L, and for TSS = 0.65 mg/L).

DESeq2 was used to identify zOTUs significantly (adjusted *p*-value ≤ 0.01) contributing to the observed shifts in the bacterial community of the *Sargassum* spp. biofilm between the ‘high’ vs. ‘low’ abiotic factor groupings. Spearman correlation coefficients were calculated using the microbiomeSeq package to link the most dominant taxa in *Sargassum* spp. biofilms to the relevant abiotic factors. Correlations were illustrated as heatmaps, and *p*-values were corrected for multiple comparisons using the Benjamini-Hochberg method.

## 3. Results

### 3.1. Variation in the Biofilm Community along the Growth-Reproduction-Senescence Cycle

**Figure 1:**
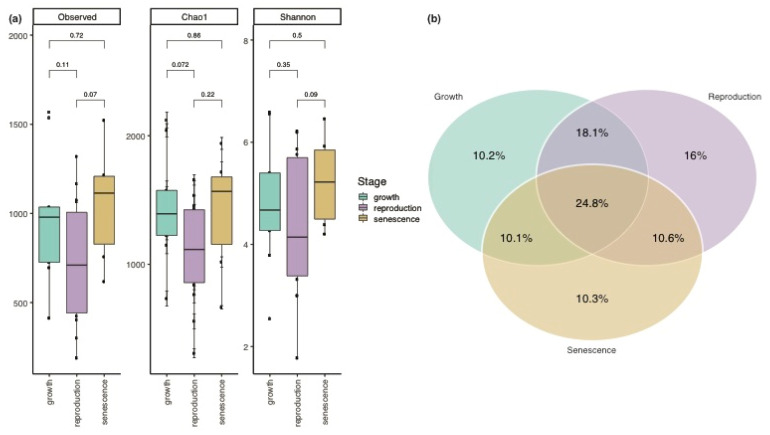
Alpha diversity of *Sargassum* spp. biofilm samples. (**a**) Observed richness, estimated richness (Chao1) and Shannon index of microbial communities associated with *Sargassum* spp. biofilm samples collected along the annual growth-reproduction-senescence cycle. A Student’s t-test was applied to compare means of life stages (‘growth’ vs. ‘reproduction’, ‘reproduction’ vs. ‘senescence’, and ‘growth’ vs. ‘senescence’), however, no significant differences in the alpha diversity measures were observed (*p*-values given above brackets). (**b**) Percentages of shared and unique zOTUs associated with the macroalgal biofilm samples from ‘growth’, ‘reproduction’, and ‘senescence’ stages are represented as Venn diagram.

**Figure 2:**
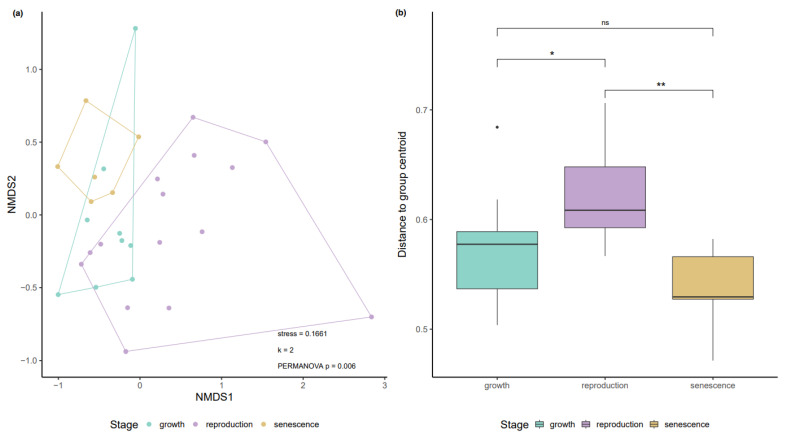
Microbial community structure and dispersion of the *Sargassum* spp. biofilm. (**a**) Non-metric multidimensional scaling (NMDS) based on Bray–Curtis dissimilarities showing a shift in the microbial community composition along the annual growth-reproduction-senescence cycle of *Sargassum* spp. (**b**) Microbial community dispersion, measured as distance to the group centroid, indicates a higher heterogeneity in the biofilm-associated microbial community during the ‘reproduction’ stage of *Sargassum* spp. A Student’s t-test was performed for pairwise comparison (ns = not significant, * = *p*-value < 0.05, ** = *p*-value < 0.01; *p*-values given above square brackets).

A total of 201,818 reads were obtained for the 16S rRNA amplicon sequencing dataset of the 30 *Sargassum* spp. biofilm samples corresponding to 8483 zero-radius operational taxonomic units (zOTUs). Following the removal of singletons, a total of 6301 unique zOTUs remained. Alpha diversity estimates of the bacterial community associated with the *Sargassum* spp. biofilm remained stable throughout the three different life stages of *Sargassum* spp. (Figure 1a). However, the high number of unique zOTUs (total of 75.2%) associated with the three life stages suggests that the community composition changes along the annual growth-reproduction-senescence cycle. Only 24.8% of the zOTUs were present in at least one of the samples from all three life stages (Figure 1b). The ‘reproduction’ stage had the highest percentage (16%) of unique zOTUs, followed by almost identical abundances of unique zOTUs in the ‘growth’ (10.2%) and ‘senescence’ (10.3%) stage. non-metric multidimensional scaling (NMDS) indicates a shift in the microbial community structure along the annual growth-reproduction-senescence cycle, which was further supported by a permutational multivariate analysis of variance (PERMANOVA: F_(2,27)_ = 1.554, *p*-value = 0.006, R2 = 0.103; Figure 2a). However, this result might have been affected by a significant variation in the sample dispersion (PERMDISP: F_(2,27)_ = 8.309, *p*-value = 0.001). Biofilm samples from the ‘reproduction’ stage showed a significantly higher dispersion around their group centroid when compared to samples from the ‘growth’ and ‘senescence’ stage (Figure 2b).

**Figure 3:**
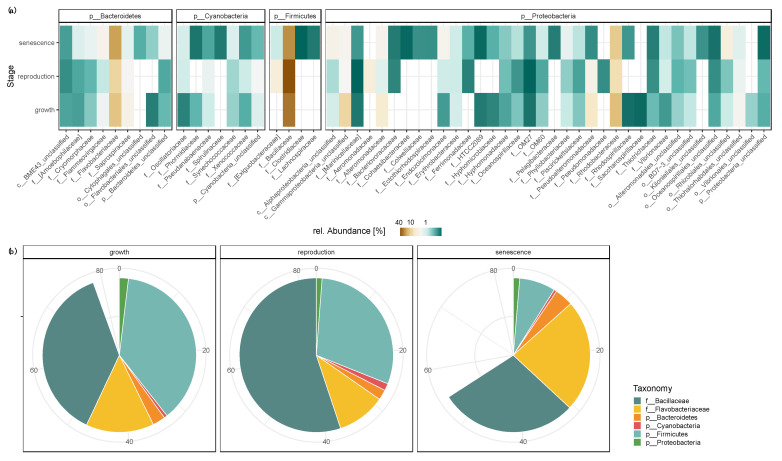
Dominant taxonomic groups associated with the biofilm of *Sargassum* spp. (**a**) Mean relative abundances of top bacterial families (mean relative abundance > 0.1%) belonging to the most dominant bacterial phyla (i.e., *Bacteroidetes*, *Cyanobacteria*, *Firmicutes*, and *Proteobacteria*) associated with the macroalgal biofilm throughout the annual growth-reproduction-senescence cycle of *Sargassum* spp. When bacterial family (**f**) is unclassified/unknown, the lowest possible taxonomic rank is given: p-phylum, c-class, o-order. (**b**) Total mean relative abundance of the top four bacterial phyla (i.e., *Bacteroidetes*, *Cyanobacteria*, *Firmicutes*, and *Proteobacteria*) and the two most abundant bacterial families, *Bacillaceae* and *Flavobacteriaceae*, belonging to the phyla *Firmicutes* and *Bacteroidetes*, respectively.

The most abundant bacterial families found on *Sargassum* spp. (Figure 3a) were *Bacillaceae* (annual average 35% ± 5.9% SD), *Flavobacteriaceae* (annual average 12% ± 0.6% SD) and *Rhodobacteraceae* (annual average 10% ± 1.0% SD). Throughout the sampling regime, *Bacillaceae* was the most dominant bacterial family associated with *Sargassum* spp. biofilm samples with an up to two-fold decrease in abundance during the ‘senescence’ stage (24.1% ± 0.8% SD in the ‘senescence’ stage versus 31.1% ± 10.4% SD and 45.8% ± 30.1% SD in the ‘growth’ and ‘reproduction’ stage, respectively; Figure 3b). *Flavobacteriaceae* displayed the opposite trend, with an up to two-fold increase in the relative abundance during the ‘senescence’ stage (19.5% ± 8.3% SD) compared to the ‘growth’ (11.9% ± 5.3% SD) and ‘reproduction’ (8.5% ± 6.4% SD) stages (Figure 3b).

### 3.2. Influence of Abiotic Factors

Analysis of collinearity between the abiotic factors led to the exclusion of the following variables: particulate organic carbon (POC), chlorophyll *a* (Chl a), phosphate (PO_4_^3−^), the sum of nitrite and nitrate (NO_2_^−^-NO_3_^−^), and nitrite (NO_2_^−^; Figure S3). A principal component analysis (PCA), including non-purgeable organic carbon (NPOC), ammonium (NH_4_^+^), temperature, silica (SiO_2_), total suspended solids (TSS), non-purgeable inorganic carbon (NPIC), and particulate nitrogen (PN), revealed that the most relevant abiotic factors responsible for overall variation amongst sampling dates were NPIC, temperature, TSS, PN, SiO_2_, and NPOC (Figure S4) with the first two principal components explaining 67.9% of the variation between samples.

**Figure 4:**
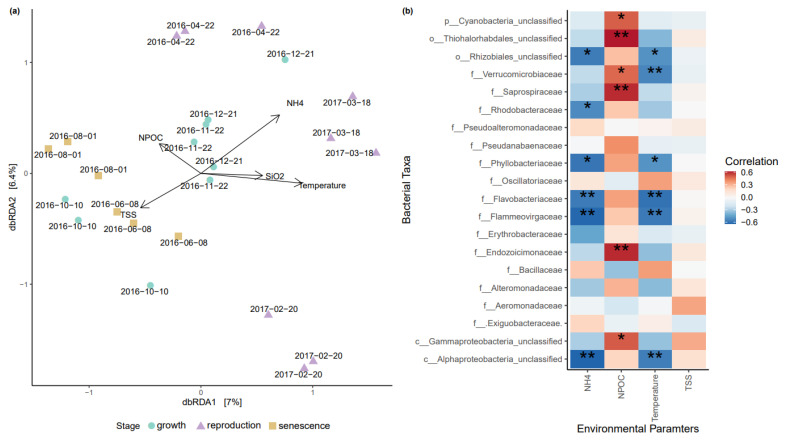
Environmental factors structuring the *Sargassum* spp. biofilm community. (**a**) Distance-based redundancy analysis (db-RDA) based on Bray–Curtis dissimilarities displaying the effect of ammonium (NH_4_^+^), temperature, silica (SiO_2_), total suspended solids (TSS), and non-purgeable organic carbon (NPOC) on the microbial community composition of the *Sargassum* biofilm. (**b**) Spearman rank correlation of top bacterial families with fluctuations in key abiotic factors (NH_4_^+^, NPOC, temperature, TSS). Strength of correlation indicated by color gradient, with significant correlations highlighted using asterisks (* for *p*-value < 0.05, ** for *p*-value < 0.01). When bacterial family (f) is unclassified/unknown, the lowest possible taxonomic rank is given: p-phylum, c-class, o-order.

A distance-based Redundancy Analysis (db-RDA) was conducted testing the abiotic influence on *Sargassum* spp. associated zOTUs. The *Sargassum* spp. biofilm samples showed a clear clustering among the different sampling time points with TSS, NH_4_^+^, SiO_2_, and NPOC as significant abiotic factors (Figure 4a and Table S1). A variance partitioning analysis suggested that the abiotic factors explained 29% of microbial community variation for *Sargassum* spp. zOTUs.

Based on the db-RDA model, a community correlation heatmap was generated, highlighting the significant correlation (*p*-value < 0.05) between bacterial taxa and the abiotic factors of NH_4_^+^, temperature, SiO_2_, TSS and NPOC (Figure 4b). For example, the NH_4_^+^ concentration and seawater temperature were significantly correlated with a reduction in bacterial taxa belonging to *Rhizobiales*, *Phyllobacteriaceae*, *Flavobacteriaceae*, *Flammeovirgaceae*, and *Alphaproteobacteria* (Figure 4b). Bacterial families such as *Rhodobacteraceae* (NH_4_^+^) and *Verrucomicrobiaceae* (temperature), however, were only significantly correlated with one of these two abiotic factors (Figure 4b). *Cyanobacteria*, *Thiohalorhabdales*, *Verrucomicrobiaceae*, *Saprospiraceae*, *Endozoicimonaceae*, and *Gammaproteobacteria* were positively correlated with an increase in NPOC concentrations (Figure 4b). TSS was not significantly correlated to any of the bacterial families (Figure 4b).

**Figure 5:**
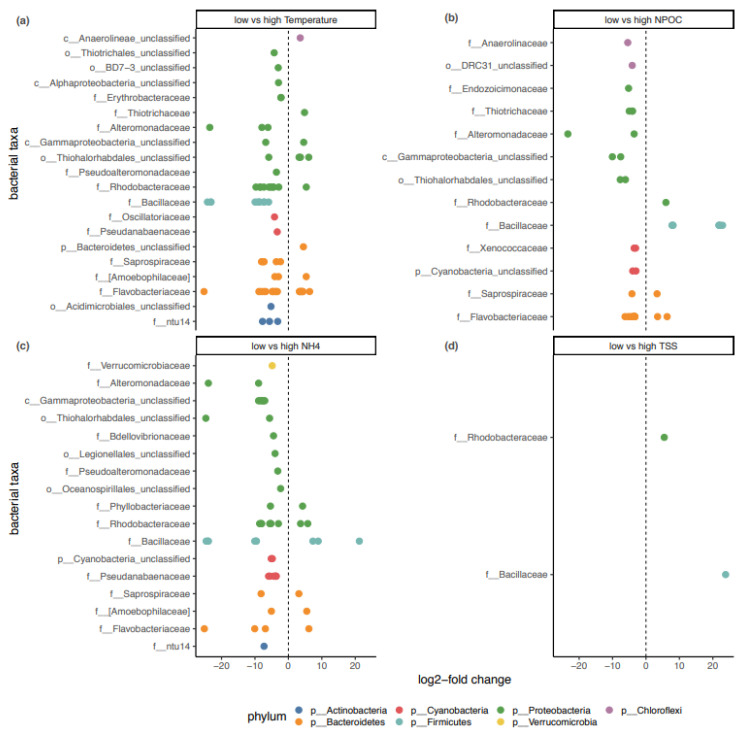
Bacterial taxa significantly enriched with fluctuations in (**a**) temperature, (**b**) non-purgeable organic carbon (NPOC) concentrations, (**c**) ammonia (NH4) concentrations, and (**d**) total suspended solid (TSS) concentrations (from top left to bottom right). Positive values of log2-fold change indicate a significant increase in the zOTU relative abundance. FDR-adjusted *p*-value was set to a threshold of *p*-value ≤ 0.01. When bacterial family (f) is unclassified/unknown, the lowest possible taxonomic rank is given: p-phylum, c-class, o-order.

The differential expression analysis (DESeq2) and community correlation heatmap shared several bacterial families, and in some instances, the DESeq2 results corroborated patterns observed in the correlation heatmap (Figure 5, FDR-adjusted *p* values ≤ 0.01). In total, 42% of the zOTUs contributing to the observed differences between low and high temperature groups belonged to the *Rhodobacteraceae* and *Flavobacteriaceae* families. Furthermore, 27% of the zOTUs causing statistical differences between low NH_4_^+^ and high NH_4_^+^ groups also belonged to *Rhodobacteraceae* and *Flavobacteriaceae*. Between 14% and 17% of the zOTUs with statistical differences between treatment groups of the abiotic factors belonged to *Bacillaceae* (Figure 5).

### 3.3. Metabolic Functions of Metagenome Assembled Genomes

A total of 20 metagenome-assembled genomes (MAGs) were included in the analysis belonging to the bacterial phyla *Firmicutes* (six MAGs), *Chloroflexota* (one MAG), *Actinobacteriota* (one MAG), *Cyanobacteria* (one MAG), *Bacteroidota* (four MAGs), *Verrucomicrobiota* (one MAG), *Spirochaetota* (one MAG), and *Proteobacteria* (two alphaproteobacterial MAGs and one gammaproteobacterial MAG). The taxonomic affiliation of MAGs is based on the Genome Taxonomy Database (GTDB); hence, taxonomic names vary slightly between zOTUs and MAGs. A detailed overview of the taxonomic affiliation, the completeness and contamination, as well as the relative abundance and representativeness of each individual MAG is provided in Table S2 and Figures S1 and S2.

**Figure 6:**
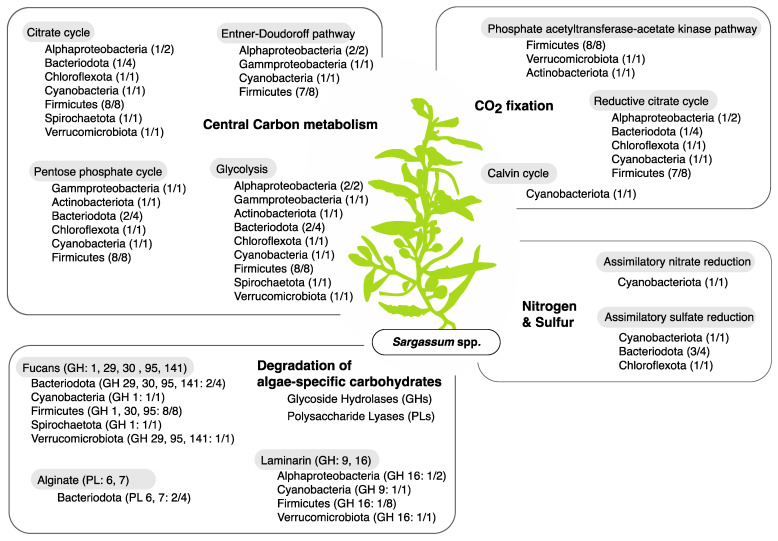
Functional overview of metagenome-assembled genomes (MAGs) associated with the *Sargassum* spp. biofilm. MAGs were recovered from a subset of samples collected during the August 2016 (n = 3; senescence stage) and February 2017 (n = 3; reproduction stage) sampling occasions. Ratios indicate the number of MAGs from each bacterial phylum where at least 60% of all KEGG Orthologous (KOs) of a KEGG module were present (e.g., 1/2 = one out of two MAGs). The genomic potential to degrade algae-specific carbohydrates was assessed using the CAZy database. Glycoside Hydrolase (GH) and Polysaccharide Lyase (PL) families specialized on the degradation of fucans, alginate, and laminarin were detected. Numbers indicate GH and PL families (e.g., GH: 1); ratios indicate the number of MAGs from each bacterial phylum genomically equipped with the putative algae-degradation capability (e.g., 1/2 = one out of two). The taxonomic affiliation of MAGs is based on the Genome Taxonomy Database (GTDB).

In total, three different carbon fixation pathways (Calvin cycle, reductive citrate cycle, and the phosphate acetyltransferase-acetate kinase pathway) were detected in 17 out of the 20 MAGs (Figure 6 and Figure S5). All genes, including the key genes rbcL (K01601) and rbcS (K01602), involved in the carbon fixation via the Calvin cycle were present in the *Cyanobacteria* MAG (Figure S6). Few genes involved in the nitrogen and sulfur metabolism were detected in the *Sargassum* spp. biofilm MAGs. However, the cyanobacterial MAG possessed 100% of the genes involved in the assimilatory nitrate reduction (Figure 6 and Figure S7). All genes for assimilatory sulfate reduction were detected in three *Bacteroidota* MAGs, one *Chloroflexota* MAG and the cyanobacterial MAG (Figure 6 and Figure S8).

In total, four distinct pathways involved in the central carbohydrate metabolism were found in 19 out of 20 MAGs (Figure 6 and Figure S9). Glycolysis was the most common and complete carbohydrate metabolism, followed by the citrate cycle, the Pentose phosphate cycle and the Entner–Doudoroff pathway. All genes for the glycolosis pathway were found in the Firmicutes MAGs, the *Chloroflexota* MAG, the *Spirochaetota* MAG and the *Verrucomicrobiota* MAG. The gammaproteobacterial MAG contained all genes involved in the Entner-Doudoroff pathway, whereas one of the *Bacteroidota* MAGs and the *Cyanobacteria* MAGs possessed all genes involved in the pentose phosphate cycle.

## 4. Discussion

Macroalgae proliferation in coral reefs is often considered a sign of ecosystem degradation [1,2,3]. The increase in macroalgae on the reef benthos fuels the algae-derived DOC pool in the water column, which triggers the growth of copiotrophic and potentially pathogenic microbes in the seawater [8,9,11]. Although the effect of macroalgae proliferation and concomitant microbialization of reef systems has been documented for some coral reefs, comparatively little is known regarding the microbial dynamics on the surface of reef-dominating macroalgae [18]. This study provides a detailed overview of the compositional and functional dynamics of the bacterial community associated with the surface biofilm of *Sargassum* spp., a dominant macroalgae at many inshore reef locations on the Great Barrier Reef (GBR) in Australia. Here, we identify shifts in the bacterial community in correlation with key abiotic factors from the ambient seawater, such as temperature, ammonium and dissolved organic carbon concentration, and shifts in the relative abundance ratios of two dominant bacterial phyla (*Firmicutes* and *Bacteroidetes*) along the annual growth-reproduction-senescence cycle of the *Sargassum* macroalgal host.

Macroalgae surfaces offer a nutrient-rich niche in contrast to the more oligotrophic water column [15,66] and hence appear to support the proliferation of a distinct microbial community [50]. The macroalgal biofilm community was dominated by *Firmicutes* (37%), *Proteobacteria* (35%) and *Bacteroidetes* (18%). These bacterial phyla are highly abundant members of the microbiome associated with various *Sargassum* species as well as with other brown algae [24,32,67,68,69,70]. In contrast, *Planctomycetes*, which were previously reported as a dominant phylum of *Sargassum* biofilms [32,68,71], represented less than one percent of the bacterial component of the biofilm in this study. Differences to previously described dominant microbial taxa might indicate an effect of geographical variation and/or host species specificity of epiphytic bacterial communities associated with brown algae.

Over the course of one year, the *Sargassum* spp. biofilm undergoes compositional shifts (Figure S8). Similar to the Mediterranean algae *Cystoseira compressa* [31], these compositional changes are successional and do not demonstrate a drastic reorganization of the biofilm-associated bacterial community. Shifts in the relative abundances of individual bacterial families belonging to the phyla *Firmicutes* and *Bacteroidetes* (i.e., *Bacillaceae* and *Flavobacteraceae*, respectively) were observed throughout the 13-month sampling period (Figure 3). Changes in the bacterial community composition of the *Sargassum* spp. biofilm significantly correlated with fluctuations in temperature, NPOC and NH_4_^+^ concentrations of the surrounding water column (Figure 4 and Figure 5). The observed effect of environmental factors on the macroalgal biofilm composition is congruent with previous studies [24,26,29]. The abiotic seawater factors included in this study (i.e., temperature, NPOC, NH_4_^+^, TSS, and SiO_2_) resolved 29% of the observed variation in the microbial community at *Sargassum* spp. surface biofilm (Figure 4 and Figure 5). Hence, as the majority of the observed microbial community shifts remain unexplained by the abiotic factors from the surrounding environment included in this study, other drivers such as host intrinsic factors (e.g., photosynthetic rate) and/or interspecific interactions of microbes must be at play and remain to be determined.

A shift in the *Firmicutes*-to-*Bacteroidetes* ratio was especially pronounced along the annual growth-reproduction-senescence cycle of *Sargassum*. For example, the phylum *Bacteroidetes* (dominated by *Flavobacteriaceae*) reached its peak relative abundance (*Firmicutes*-to-*Bacteroidetes* ratio = 0.9:1) during the senescent stage (June-August, Figure 3b) when *Sargassum* spp. sheds its fronds, decay occurs, and biomass is depleted to its minimum [41,42,43]. The macroalgae-associated *Bacteroidota* MAGs (family *Flavobacteriaceae*) recovered from the *Sargassum* biofilm samples were equipped with polysaccharide lyases (PLs) and glycoside hydrolases (GHs), putatively involved in degrading algae-derived compounds such as fucans and alginate (Figure 6). Members of the phylum *Bacteroidota* have recently been shown to be enriched in coral microbiomes and bacterioplankton communities of macroalgae-dominated reefs [51,72,73]. Furthermore, free-living members of the bacterial phylum *Bacteriodota* are known to be major responders to phytoplankton blooms due to their ability to degrade high molecular grade organic matter [74]. Hence, the increase in *Flavobacteriaceae* during the ‘senescence’ stage and their genomic potential to degrade algae-derived compounds suggests that *Flavobacteriaceae* might play a key role in the tissue degradation of the *Sargassum* spp. fronds. In contrast, during the active ‘growth’ stage (October-December) and the ‘reproduction’ stage (February-April), the *Firmicutes* (dominated by *Bacillaceae*) were 1.7 to 3.9 times more abundant, respectively, than *Bacteroidota* (Figure 3b). Although the overall relative abundance of *Bacillaceae* did not correlate with environmental fluctuations (Figure 4b), individual zOTUs affiliated with this family responded to changes in the environment (i.e., temperature, NPOC, NH_4_^+^ and TSS concentrations), suggesting a specific response of individual *Bacillaceae* members (Figure 5).

A role of the *Firmicutes*-to-*Bacteroidetes* ratio in the growth-reproduction-senescence cycle of *Sargassum* spp. has previously been suggested [51]; however, only two time points were compared. Here, we confirm the previously suggested relative abundance changes using a monthly sampling regime throughout the annual growth-reproduction-senescence cycle of *Sargassum*. Interestingly, carbohydrate composition has been reported to vary throughout the annual growth cycle of brown algae, with increased abundances of laminarin during the rapid growth phase [75,76]. Members of the phylum *Firmicutes* with the putative ability to degrade laminarin were highly enriched from October to April (Figure 3, Figure 4 and Figure 5), when *Sargassum* undergoes its active growth (October-December) and reproduction (February-April) and reaches its maximum biomass [45,46]. This suggests that variations in algae-specific carbohydrate availability might play a vital role in the observed relative abundance shifts between *Firmicutes* and *Bacteroidetes*. Hence, investigating the microbial interactions in relation to algae-derived carbohydrates throughout the growth-reproduction-senescence cycle of *Sargassum* spp. will provide valuable insights in the successional processes of the biofilm microbiome associated with a dominant macroalgae in coral reef ecosystems. It remains to be determined how changes in successional processes on the *Sargassum* microbial biofilm may affect the surrounding water column and the DDAM feedback loop. One hypothesis of future interest is that heterotrophic bacteria (such as *Firmicutes* and *Bacteroidetes*) inhabiting the surface of macroalgal primary producers may play a relevant role in regulating the DDAM feedback loop, either by consuming leaking carbohydrates or by actually facilitating their release into the water column.

## Data Availability

16S rRNA and metagenome sequences, metadata and protocols are available at the Bioplatforms Australia data portal under the Australian Microbiome project (https://www.bioplatforms.com/australian-microbiome/). Full usage requires free registration. To search for the 16S rRNA sequencing data, navigate to ‘Processed data’, select ‘Amplicon is 27f519r_bacteria’ and ‘Environment is Marine’. To search for the *Sargassum* spp. samples, add an additional contextual filter, select ‘Sample Site Location Description from the dropdown menu and search for ‘Geoffrey Bay’ and ‘Sample Type is Seaweed’. Metagenome assembled genomes (MAGs) used in this study have been deposited under NCBI BioProject PRJNA594068. Water temperature data of the sampling site (Geoffrey Bay 2 m depth) during the collection period (2016–2017) was extracted from the data repository of the Australian Institute of Marine Science (AIMS; https://data.aims.gov.au).

## Supplementary Materials

The following are available online at https://www.mdpi.com/article/10.3390/life11111199/s1, Figure S1: Relative abundances of MAGs, Figure S2: Microbial community profile based on marker genes, Figure S3: Correlation matrix for environmental parameters, Figure S4: Principal component analysis based on environmental parameters, Figure S5: Heatmap displaying the completeness of carbon fixation pathways in recovered MAGs, Figure S6: Copy number of genes associated with the Calvin Cycle, Figure S7: Heatmap displaying the completeness of the nitrogen metabolism in MAGs, Figure S8: Heatmap displaying the completeness of the sulfur metabolism in MAGs, Figure S9: Heatmap displaying the completeness of the central carbohydrate metabolism in MAGs, Table S1: Statistical output of the permutational analysis of variance for abiotic factors of the distance-based redundancy analysis, Table S2: Taxonomic affiliation, completeness, contamination and genome size of MAGs.
