# Supplementary material for: Microbial Surface Biofilm Responds to the Growth-Reproduction-Senescence Cycle of the Dominant Coral Reef Macroalgae Sargassum spp
Source: Life (Basel). 2021 Nov 6;11(11):1199. doi: 10.3390/life11111199 (PMC8621314; doi:10.3390/life11111199)
Supplement: Supplementary file 1 [file life-11-01199-s001.zip › life-1414274-supplementary.pdf]

life

MDPI

Supplementary Materials: Microbial Surface Biofilm Responds to the Growth-Reproduction-Senescence Cycle of the Dominant Coral Reef Macroalgae Sargassum spp.

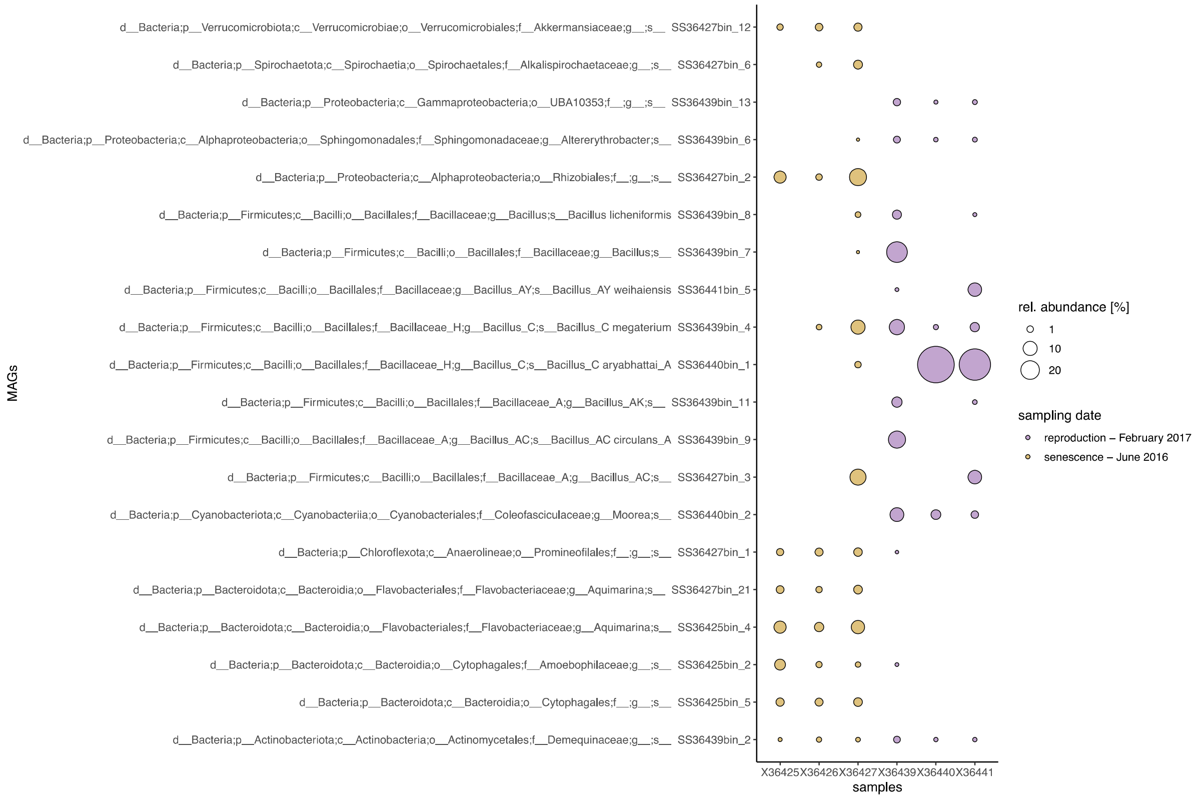

Figure S1. Relative abundances of previously reconstructed metagenome assembled genomes (MAGs) from the *Sargassum* spp. biofilm [51]. Adapter-trimmed metagenomic reads, originating from *Sargassum* spp. biofilm samples collected in June 2016 ('senescence' stage) and February 2017 ('reproduction' stage), were mapped against 20 high quality MAGs with coverM v0.2.0 (https://github.com/wwood/CoverM) using a 75% minimum alignment and 95% minimum identity threshold.

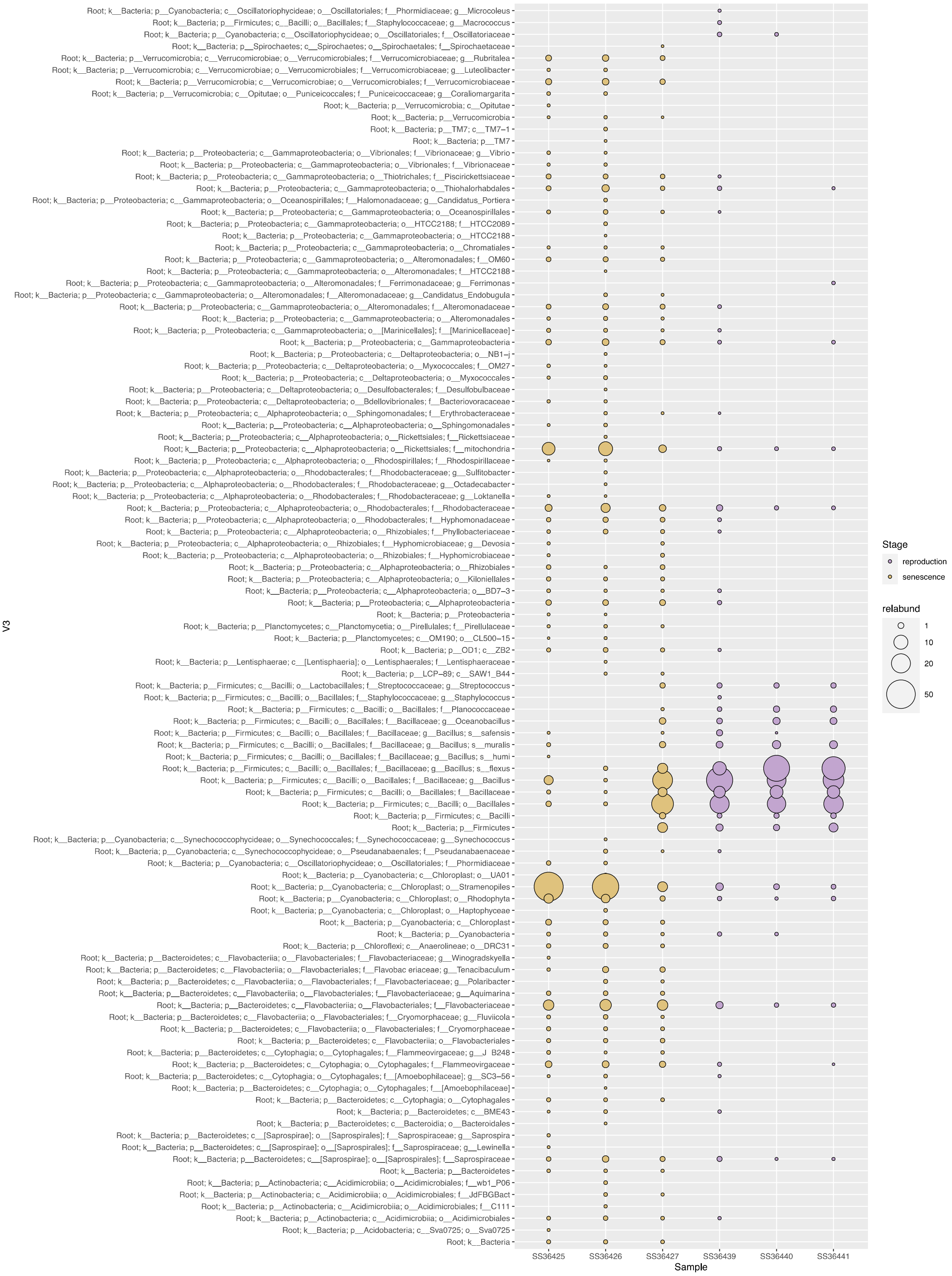

Figure S2. Overview of the microbial community profile confirms the representativeness of the retrieved metagenome assembled genomes (MAGs). The microbial community profiles were generated by extracting 16S rRNA gene fragments of adapter-trimmed metagenome reads using GraftM v0.12.0, (https://github.com/geronimp/graftM). The bubbleplot displays only the microbial taxa with a relative abundance >0.1%.

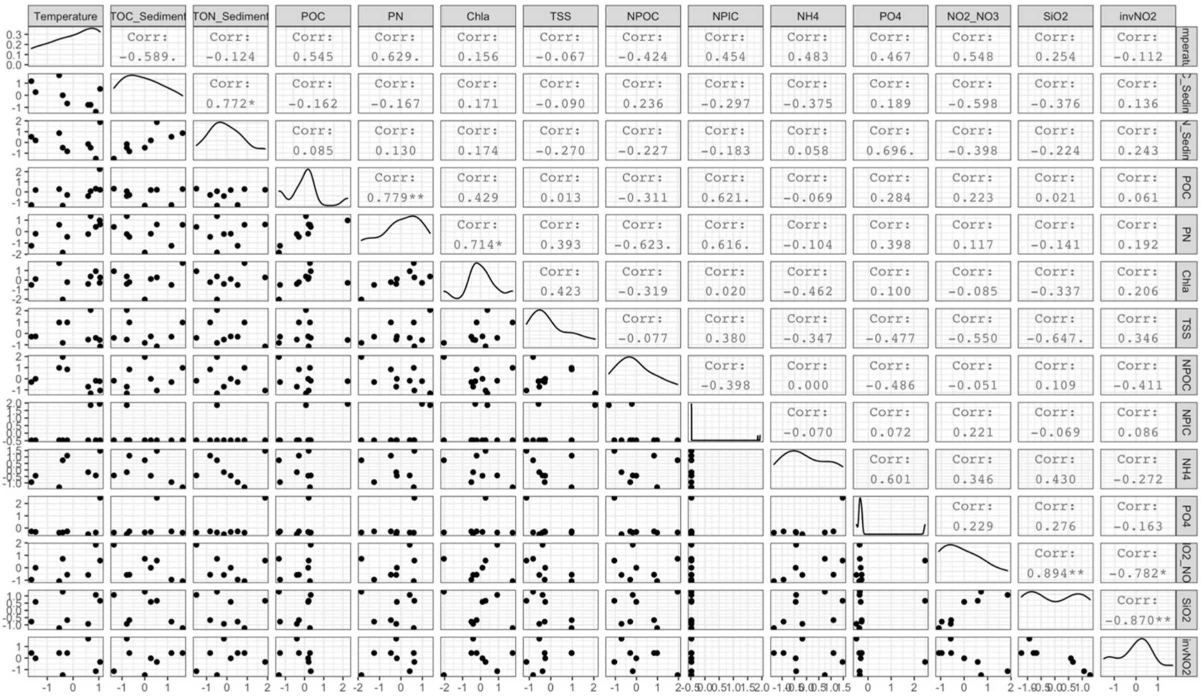

Figure S3. Correlation matrix for environmental parameters collected at Geoffrey Bay computed with Pearson correlation. Correlation threshold set to values equal to or above 0.7 or values equal to or below −0.7. Values meeting these criteria are marked with asterisks.

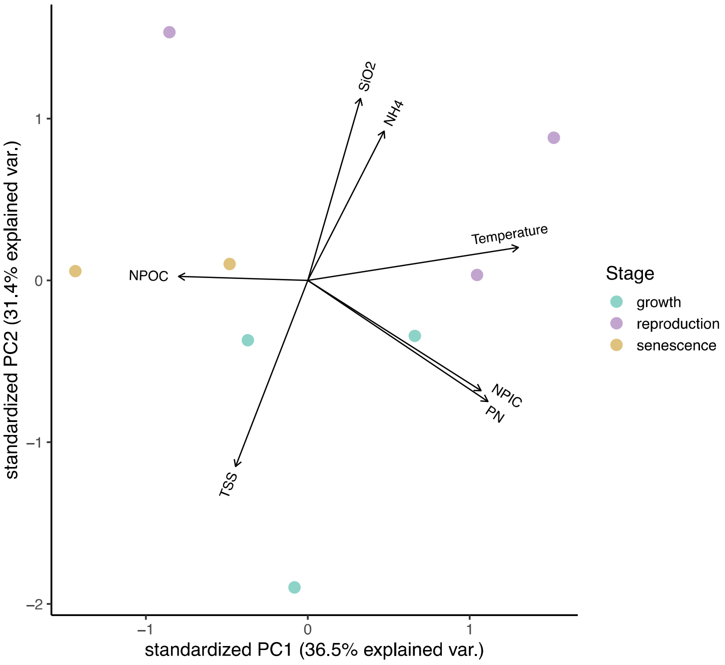

Figure S4. Principal Component Analysis (PCA) of environmental metadata displaying seawater parameters that significantly vary between sampling time points. Color indicates the growth-reproduction-senescence stage of the host. The selected seawater parameters include: NPOC = non-purgeable organic carbon, TSS = total suspended solids, PN = particulate nitrogen, NPIC = non-purgeable inorganic carbon, SiO2 = Silica, NH4 = ammonium concentration, and seawater temperature.

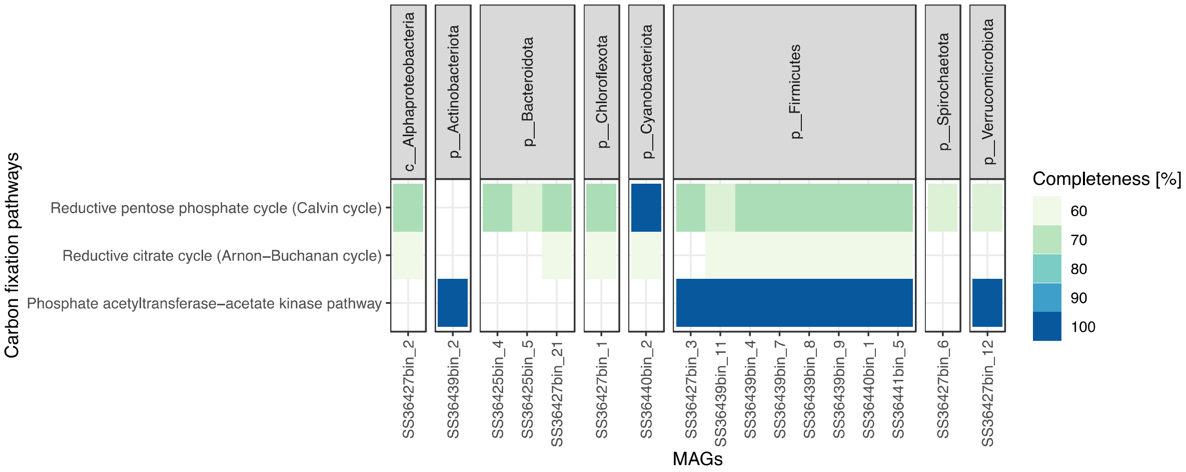

Figure S5. Carbon fixation pathways detected in recovered metagenome assembled genomes (MAGs). Colour indicates the completeness (in percent) of KEGG modules.

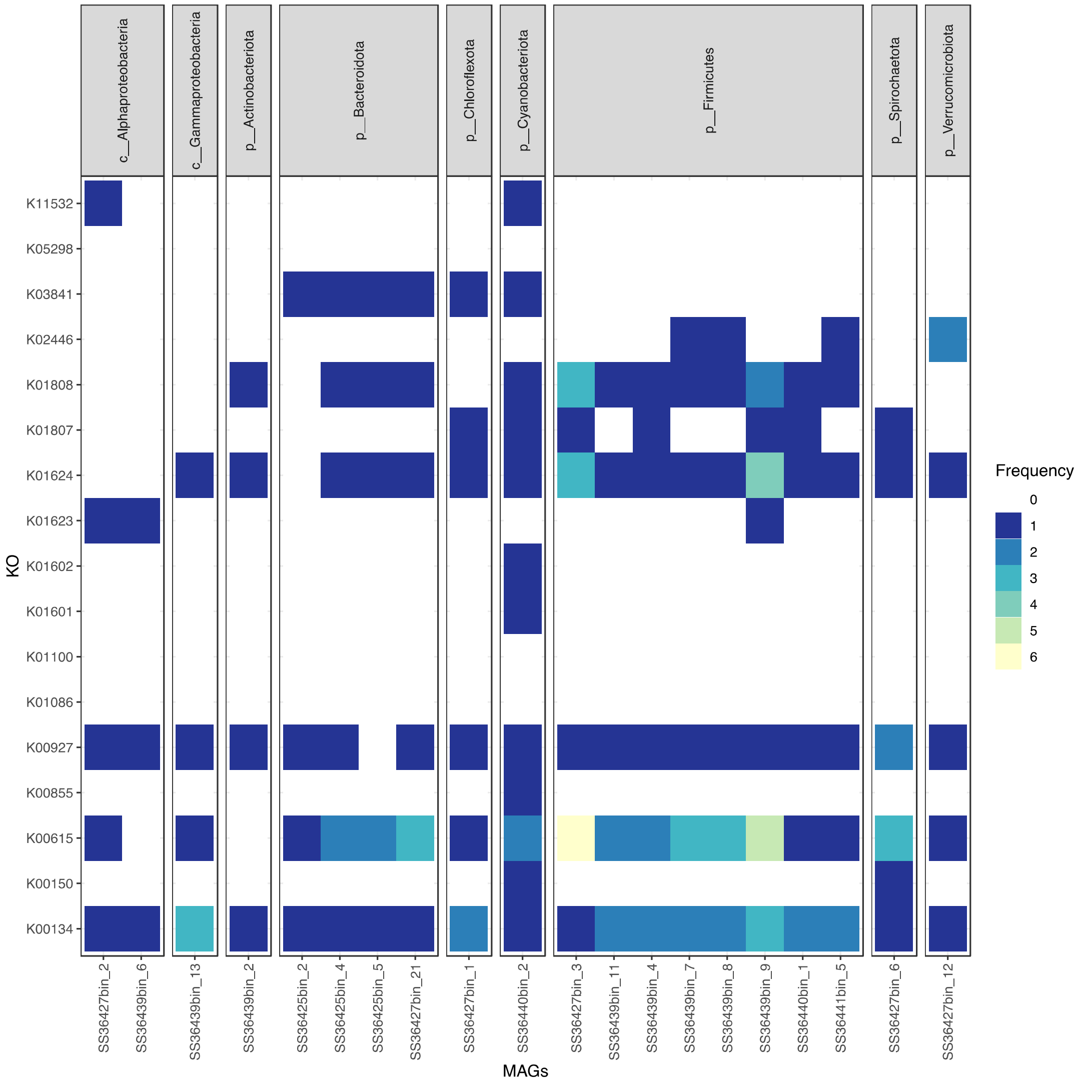

Figure S6. Copy number (frequency) of annotated KEGG KOs associated with autotrophic carbon fixation via the Calvin Cycle for recovered metagenome assembled genomes (MAGs).

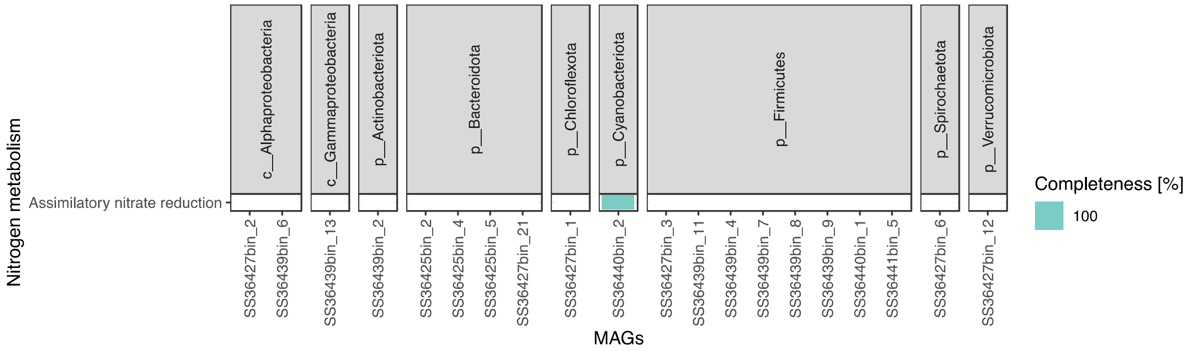

Figure S7. Nitrogen related KEGG modules detected in recovered metagenome assembled genomes (MAGs). Colour indicates the completeness (in percent) of KEGG modules.

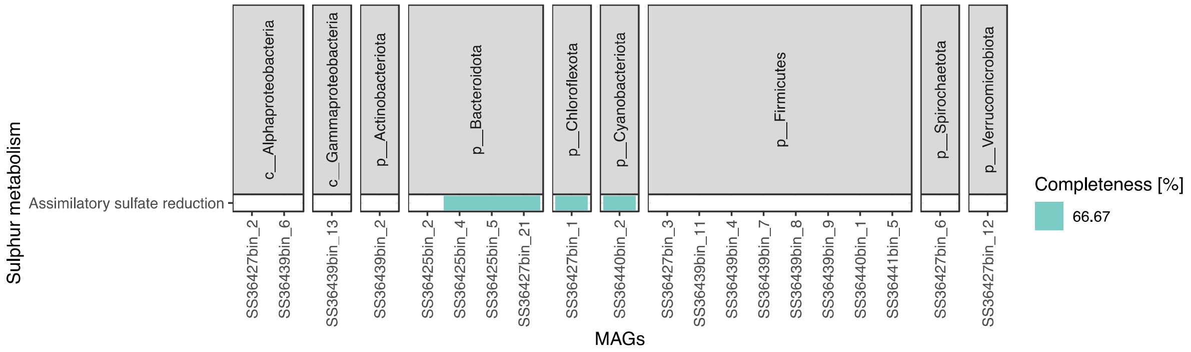

Figure S8. Sulphur related KEGG modules detected in recovered metagenome assembled genomes (MAGs). Colour indicates the completeness (in percent) of KEGG modules.

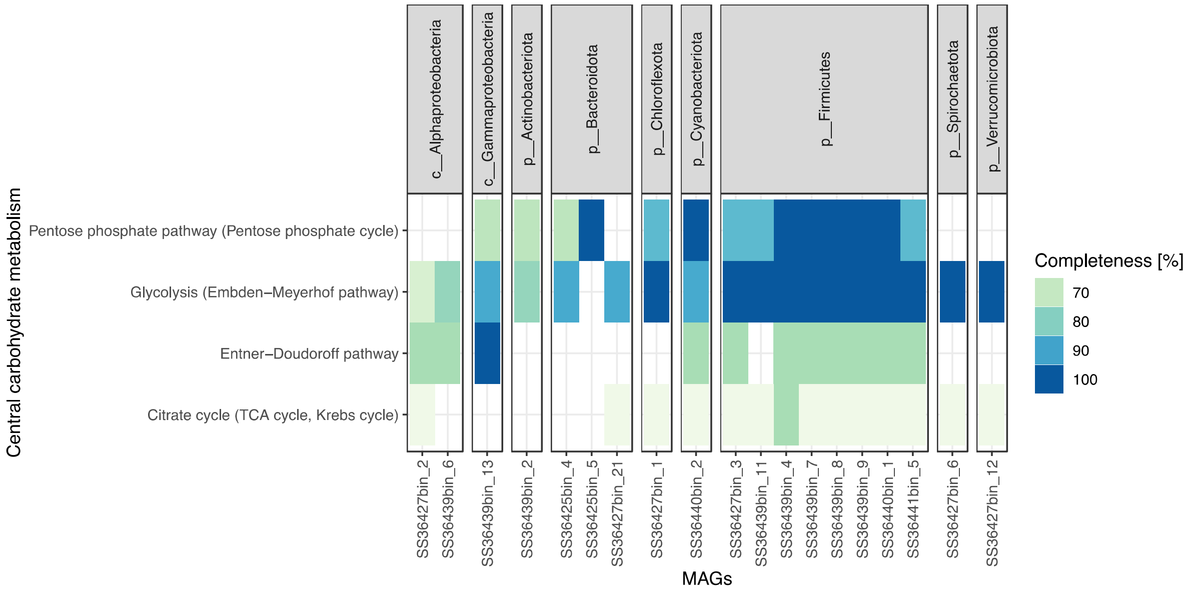

Figure S9. Central carbohydrate metabolism related KEGG modules detected in recovered metagenome assembled genomes (MAGs). Colour indicates the completeness (in percent) of KEGG modules.

Table S1. Permutational Analysis of Variance for abiotic factors of the distance-based Redundancy Analysis (db-RDA) significantly explaining observed compositional variations of the *Sargassum* spp. biofilm community.

***Sargassum* sp.**					
**Variable**	**R2.adj**	**Df**	**AIC**	**F**	**Pf(<F)**
Temperature	0.02502	1	57.197	1.5917	0.002 **
NH4	0.041662	1	57.669	1.3806	0.006 **
NPOC	0.056169	1	58.132	1.3228	0.02 *
TSS	0.070895	1	58.524	1.317	0.024 *
SiO2	0.092072	1	58.673	1.4432	0.018 *

Table S2. Taxonomic affiliation, completeness, contamination and genome size of each individual metagenome assembled genome (MAG) retrieved from *Sargassum* spp. biofilm samples.

MAG ID	GTDB-tk Taxonomy	Completeness In %	Contamination In %	Genome Size (bp)
SS36425bin_2	d__Bacteria;p__Bacteroidota;c__Bacteroidia;o__Cytophagales;f__Amoebophilaceae;g__UBA8403;s__	79.4	0.55	1098163
SS36425bin_4	d__Bacteria;p__Bacteroidota;c__Bacteroidia;o__Flavobacteriales;f__Flavobacteriaceae;g__Aquimarina;s__	82.71	9.78	1865337
SS36425bin_5	d__Bacteria;p__Bacteroidota;c__Bacteroidia;o__Cytophagales;f__UBA9547;g__;s__	75.95	3	3635561
SS36427bin_1	d__Bacteria;p__Chloroflexota;c__Anaerolineae;o__Promineofilales;f__Promineofilaceae;g__;s__	98.18	3.64	5809359
SS36427bin_12	d__Bacteria;p__Verrucomicrobiota;c__Verrucomicrobiae;o__Verrucomicrobiales;f__Akkermansiaceae;g__;s__	87.33	2.38	2711003
SS36427bin_2	d__Bacteria;p__Proteobacteria;c__Alphaproteobacteria;o__Rhizobiales;f__;g__;s__	92	0.2	2305540
SS36427bin_21	d__Bacteria;p__Bacteroidota;c__Bacteroidia;o__Flavobacteriales;f__Flavobacteriaceae;g__Aquimarina;s__	78.54	3.26	2093196
SS36427bin_3	d__Bacteria;p__Firmicutes;c__Bacilli;o__Bacillales;f__Bacillaceae_A;g__Bacillus_AC;s__	92.78	0.86	5236246
SS36427bin_6	d__Bacteria;p__Spirochaetota;c__Spirochaetia;o__Spirochaetales;f__Alkalispirochaetaceae;g__;s__	93.2	0	1422575
SS36439bin_11	d__Bacteria;p__Firmicutes;c__Bacilli;o__Bacillales;f__Bacillaceae_A;g__Bacillus_AK;s__	80.78	2.03	2922325
SS36439bin_13	d__Bacteria;p__Proteobacteria;c__Gammaproteobacteria;o__UBA10353;f__UBA7415;g__UBA7415;s__	87.79	10.98	2595422
SS36439bin_2	d__Bacteria;p__Actinobacteriota;c__Actinobacteria;o__Actinomycetales;f__Demequinaceae;g__;s__	85.83	0	1084055
SS36439bin_4	d__Bacteria;p__Firmicutes;c__Bacilli;o__Bacillales;f__Bacillaceae_H;g__Bacillus_C;s__Bacillus_C megaterium	99.43	0.03	4845095
SS36439bin_6	d__Bacteria;p__Proteobacteria;c__Alphaproteobacteria;o__Sphingomonadales;f__Sphingomonadaceae;g__Altererythrobacter;s__	80.01	1.87	2395843
SS36439bin_7	d__Bacteria;p__Firmicutes;c__Bacilli;o__Bacillales;f__Bacillaceae;g__Bacillus;s__	81.03	0	3980272
SS36439bin_8	d__Bacteria;p__Firmicutes;c__Bacilli;o__Bacillales;f__Bacillaceae;g__Bacillus;s__Bacillus licheniformis	97.8	0	4078403
SS36439bin_9	d__Bacteria;p__Firmicutes;c__Bacilli;o__Bacillales;f__Bacillaceae_A;g__Bacillus_AC;s__Bacillus_AC circulans_A	92.78	2.79	5551538
SS36440bin_1	d__Bacteria;p__Firmicutes;c__Bacilli;o__Bacillales;f__Bacillaceae_H;g__Bacillus_C;s__Bacillus_C aryabhattai_A	99.43	0	3974674
SS36440bin_2	d__Bacteria;p__Cyanobacteriota;c__Cyanobacteriia;o__Cyanobacteriales;f__Coleofasciculaceae;g__Moorea;s__	98.44	0.81	7749996

SS36441bin_5	d__Bacteria;p__Firmicutes;c__Bacilli;o__Bacillales;f__Bacillaceae;g__Bacillus_AY;<br>s__Bacillus_AY weihaiensis	98.97	2.35	4791764
